# Mycobacterium immunogenum-Induced Thrombocytopenia

**DOI:** 10.7759/cureus.78306

**Published:** 2025-01-31

**Authors:** Alejandra Viera, Peter Wahba, Jannelle Vicens, Sandra Sepulveda, Marco Ruiz-Andia

**Affiliations:** 1 Medical School, Herbert Wertheim College of Medicine, Florida International University, Miami, USA; 2 Malignant Hematology/Bone Marrow Transplant/HIV Oncology, Miami Cancer Institute, Miami, USA

**Keywords:** idiopathic thrombocytopenia, immune thrombocytopenic purpura (itp), infectious disease diagnosis, lymphadenopathy, mycobacterium immunogenum, rare hematology findings

## Abstract

*Mycobacterium immunogenum *is a fast-growing non-tuberculous mycobacterium often found in soil, water, and aerosols. It primarily affects immunocompromised individuals. One common area of infection is the pulmonary system, but lymph nodes, bones, and joints are also commonly involved.

We report a case of a young female with a two-year history of lymphadenopathy who developed thrombocytopenia with petechiae, bruising, and mucosal bleeding. Initially, she had a positron emission tomography/computed tomography (PET/CT) scan showing elevated standardized uptake value concerning for malignancy, but a biopsy shortly after revealed more of a reactive follicular hyperplastic etiology. Her platelet count was severely low (3 K/uL) and a diagnosis of immune (idiopathic) thrombocytopenic purpura (ITP) was made. After numerous negative tests, a Karius test was performed for possible infectious etiology, which revealed *Mycobacterium immunogenum*. The patient responded well to intravenous immunoglobulin and was managed outpatient without complications.

This case highlights the rare association of *Mycobacterium immunogenum *with ITP. There are studies that relate the occurrence of ITP in patients with *Mycobacterium tuberculosis*, another bacterium of similar taxonomy to *Mycobacterium immunogenum.* This case report is significant because there is limited research on the complications of *Mycobacterium immunogenum*, particularly its relationship to ITP. Given the lack of information regarding this association, this report serves as a catalyst for further research into identifying potential rare causes of ITP.

## Introduction

The purpose of this case study is to document and analyze a rare clinical presentation involving a patient diagnosed with immune thrombocytopenia secondary to *Mycobacterium immunogenum* infection. By comparing the results of this study with the literature, this report aims to enhance the understanding of the pathophysiology, diagnosis, and treatment of this condition.

*Mycobacterium immunogenum* is a rapidly growing mycobacterium that was originally classified as a non-tuberculous mycobacterium [1]. This acid-fast, rod-shaped, and non-motile bacterium is commonly found in swimming pools, hospital settings, and drinking water distribution systems [2]. It is associated with a range of medical conditions, including respiratory infections in immunocompromised individuals, chronic obstructive pulmonary disease (COPD) exacerbations, hypersensitivity pneumonitis as a result of prolonged exposure, and nosocomial infections [1].

Immune thrombocytopenic purpura (ITP), also known as idiopathic thrombocytopenia, is a condition characterized by auto-antibody-mediated platelet destruction [3]. Auto-antibodies target surface antigens on platelets, such as GPIIb/IIIa and GPIb-IX-V [4]. This causes a decrease in platelet count, leading to various consequences such as epistaxis, petechiae, mucosal bleeding cuts, and, in severe cases, internal hemorrhage. This condition is also associated with splenomegaly, as the spleen sequesters and destroys platelets coated with antibodies. Thus, refractory cases of ITP are treated with splenectomy. While the cause of ITP is often unknown (hence "idiopathic"), it can be triggered by certain medications like sulfonamides, autoimmune disorders such as systemic lupus erythematosus, and specific viruses like cytomegalovirus and Epstein-Barr virus (EBV). In this case study, it is hypothesized that ITP is triggered by *Mycobacterium immunogenum* infection.

This case report is significant because there is limited research on the complications of *Mycobacterium immunogenum*, particularly regarding its relationship to ITP. This report describes a possible association between *Mycobacterium immunogenum* and ITP based on a single observed instance. Given the lack of information surrounding this association, this report serves as a catalyst for further research into identifying potential rare causes of ITP.

## Case presentation

A 33-year-old female with a history of lymphadenopathy and several non-diagnostic biopsies, iron-deficiency anemia, and splenomegaly was being followed by a hematologist for routine monitoring. A positron emission tomography/computed tomography (PET/CT) scan completed in December 2021 showed lymphadenopathy with elevated standardized uptake value (SUV) concerning for malignancy. A biopsy was performed shortly after, and the findings were consistent with reactive follicular hyperplasia. She remained on observation only.

**Figure 1:**
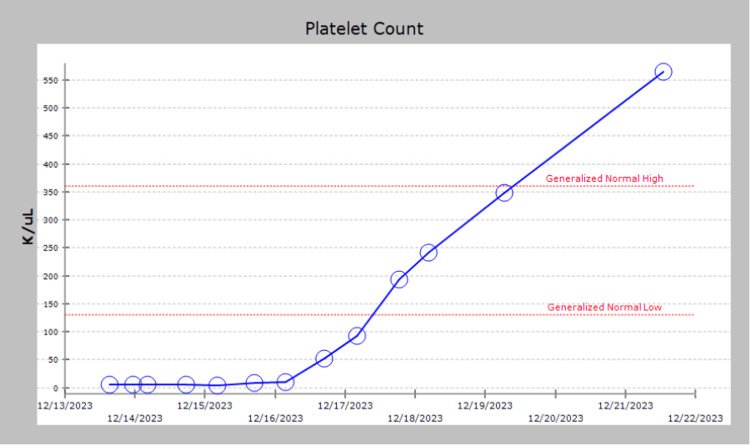
Trends of platelet counts throughout admission

In December 2023, the patient presented to the outpatient clinic because she was not feeling well. Upon physical examination, she exhibited petechiae, bruises on her arms, epistaxis, and bleeding of her gums for the past few days. Labs revealed severe thrombocytopenia with a platelet count of 3K/uL (Figure 1).

The patient was admitted for further workup. At the hospital, her initial chest X-ray was negative. Abdominal ultrasound revealed hepatomegaly, severe steatosis, and mild splenomegaly. A PET/CT scan revealed several F-fluorodeoxyglucose (FDG) avid lymph nodes with a mixed pattern of change (dating back to October 2022; multiple core biopsies of lymph nodes and bone marrow biopsies were all negative). The patient was then consulted by the infectious disease team and found to have negative hepatitis B and C serology, a negative mononucleosis test, HIV negative, syphilis negative, EBV DNA negative, negative methicillin-resistant *Staphylococcus aureus* (MRSA) nasal screening, and negative blood cultures during admission. The patient was started on pulse dexamethasone 40 mg IV daily for four days. 

Karius testing was ordered to determine potential infectious etiologies for the patient’s severe thrombocytopenia after she was diagnosed with ITP in the absence of other causative factors. The Karius test returned positive for *Mycobacterium immunogenum*. The patient was treated with two IV immunoglobulin infusions, which restored her platelet count. Given the nature of immune-mediated ITP and the otherwise unknown causative factor for severe thrombocytopenia, the patient was monitored closely with lab tests twice a week. She was referred to outpatient infectious disease management (Table 1).

**Table 1:** Diagnostic evaluation during hospital admission PET/CT: Positron emission tomography/computed tomography; FDG: F-fluorodeoxyglucose; EBV: Epstein-Barr virus; MRSA: Methicillin-resistant *Staphylococcus aureus*

Labs/imaging on admission	Results
Platelet count (K/uL)	3 (normal: 150-450)
Abdominal ultrasound	Hepatosplenomegaly, severe steatosis, mild splenomegaly
PET/CT	FDG avid lymph nodes
Hepatitis C serology	Negative
Hepatitis B serology	Negative
HIV test	Negative
Mononucleosis test	Negative
Syphilis	Negative
EBV DNA	Negative
MRSA nasal screening	Negative
Blood culture	Negative
Karius test	*Mycobacterium immunogenum *positive

Given the clinical course, we believe she had ITP secondary to *Mycobacterium immunogenum*, both of which are now entirely resolved. No additional complications have risen since then. 

## Discussion

Immune thrombocytopenia is a condition characterized by an autoimmune attack on specific surface antigens located on the platelets. This leads to a series of complications, including impaired platelet aggregation, which results in an increased risk of bleeding. This patient presented with petechiae, bruising, bleeding gums, and epistaxis. ITP is a diagnosis of exclusion and can be classified as primary or secondary. It is classified as primary when no other underlying cause is identified and secondary when a primary trigger is identified [5]. In our case, the patient was diagnosed with *Mycobacterium immunogenum* infection while presenting with ITP, leading to a diagnosis of secondary ITP.

The literature does not show a correlation between *Mycobacterium immunogenum* and the occurrence of ITP. However, there are studies that relate the occurrence of ITP in patients with *Mycobacterium tuberculosis*, another bacterium of similar taxonomy to *Mycobacterium immunogenum*. One study discussed a case of extrapulmonary tuberculosis, where the bacteria spread to the lymph nodes, causing lymphadenopathy [6]. Similar to the patient presented in this case, that patient also exhibited petechiae, gum bleeding, lymphadenopathy, and thrombocytopenia. Additionally, another case involved a patient with *Mycobacterium avium-intracellulare *infection who presented with ITP [7]. Initially, it was suspected that the ITP was secondary to HIV; however, bone marrow aspiration demonstrated atypical mycobacterium. The patient's thrombocytopenia improved with antibacterial therapy, achieving a normal platelet count by day 20 of treatment [7]. These studies, along with the case we present, suggest treating the underlying trigger improves ITP significantly and promptly.

The pathophysiology of our patient’s presentation can be explained through molecular mimicry [8]. It is hypothesized that some bacterial products resemble the receptors on the platelets, leading to antibodies that attack both the bacteria and the platelet receptors. This can result in cross-reactivity, where there is an autoimmune attack on the platelets and progression to ITP [8]. It is possible that the patient’s immune system generated antibodies against a protein from *Mycobacterium immunogenum* that also resembled components of the patient’s platelets, resulting in autoimmune destruction. Furthermore, this supports the importance of addressing the infection primarily, as thrombocytopenia tends to improve following antibiotic treatment.

Additionally, the development of ITP in this patient can also be explained by the presence of certain risk factors. Some risk factors for the occurrence of immune thrombocytopenia include female sex, obesity, and Black ethnicity [9]. Moreover, it seems to predominantly affect women in their 30s and 40s [10]. This raises the question of whether hormones may play a role in the pathophysiology of ITP. There appears to be a correlation between estrogens and the occurrence of B-cell-mediated autoimmune diseases. This may be explained by estrogen's ability to regulate certain cytokines, which can affect the immune response and make women more likely to develop autoimmune diseases [10].

The results of this case report are significant because they establish a relationship between* Mycobacterium immunogenum* infection and the occurrence of secondary ITP. Given that there is limited literature establishing this relationship, this case enhances our understanding of the potential triggers and complications of non-tuberculous mycobacterium. This case also highlights the importance of considering infectious etiologies when searching for the underlying trigger of ITP. Additionally, the proposed mechanism of molecular mimicry and the influence of hormones can further our understanding of the etiology behind this patient’s presentation. However, a limitation of this study is the sparse evidence supporting the relationship between *Mycobacterium immunogenum* and ITP. Furthermore, no concrete pathophysiology is yet known for this relationship, indicating that further research is needed to explore this association.

## Conclusions

The complications of systemic infection with non-tuberculous mycobacterium, such as *Mycobacterium immunogenum*, are not fully understood. There is limited literature available to establish the association between *Mycobacterium immunogenum* and ITP. While this case study does not provide concrete evidence of causation, it highlights the potential association of the ITP episode with mycobacterium infection. ITP is typically a diagnosis of exclusion, meaning all other etiologies must first be exhausted. Proper identification of the underlying cause is crucial in directing the management of such cases. Therefore, this case study aims to raise awareness of the possibility of *Mycobacterium immunogenum-*induced thrombocytopenia and to encourage the consideration of this infection. Infection with non-tuberculous mycobacterium should be explored and excluded when investigating cases of idiopathic ITP, especially if more common etiologies have already been ruled out. 

Future research should focus on further exploring the pathophysiological mechanisms linking *Mycobacterium immunogenum* to ITP, including the roles of molecular mimicry and immune dysregulation. Improved diagnostic strategies, including routine infectious screening in cases of unexplained ITP, might be a useful strategy for understanding and managing such cases. 
